# Supplementary material for: Exercise Suppresses Head and Neck Squamous Cell Carcinoma Growth via Oncostatin M
Source: Cancers (Basel). 2024 Mar 18;16(6):1187. doi: 10.3390/cancers16061187 (PMC10969669; doi:10.3390/cancers16061187)
Supplement: Supplementary file 1 [file cancers-16-01187-s001.zip › cancers-2908809-supplementary.pdf]

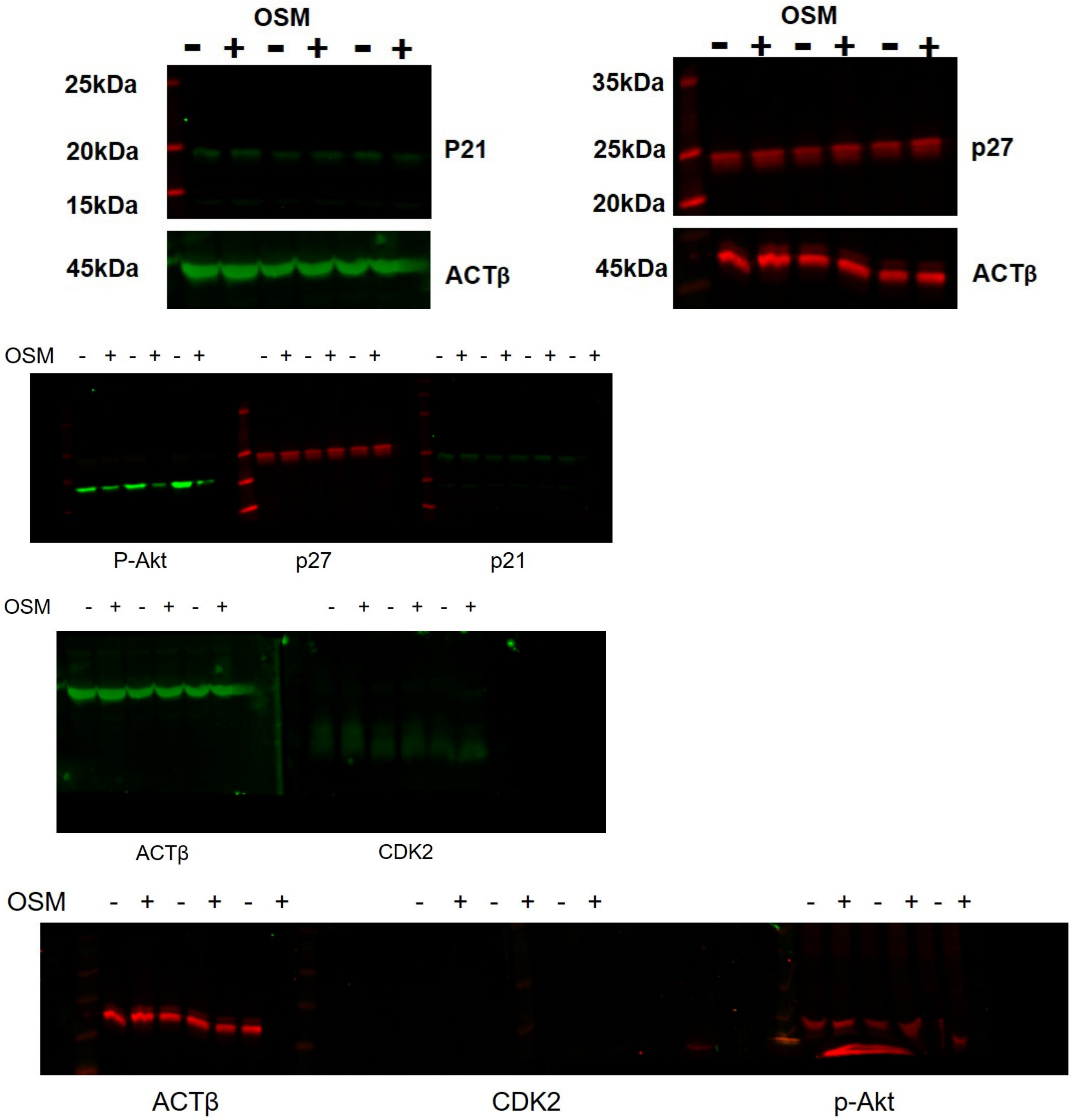

Figure S1. Correspond to the values of p21 and p27 in Figure 6.

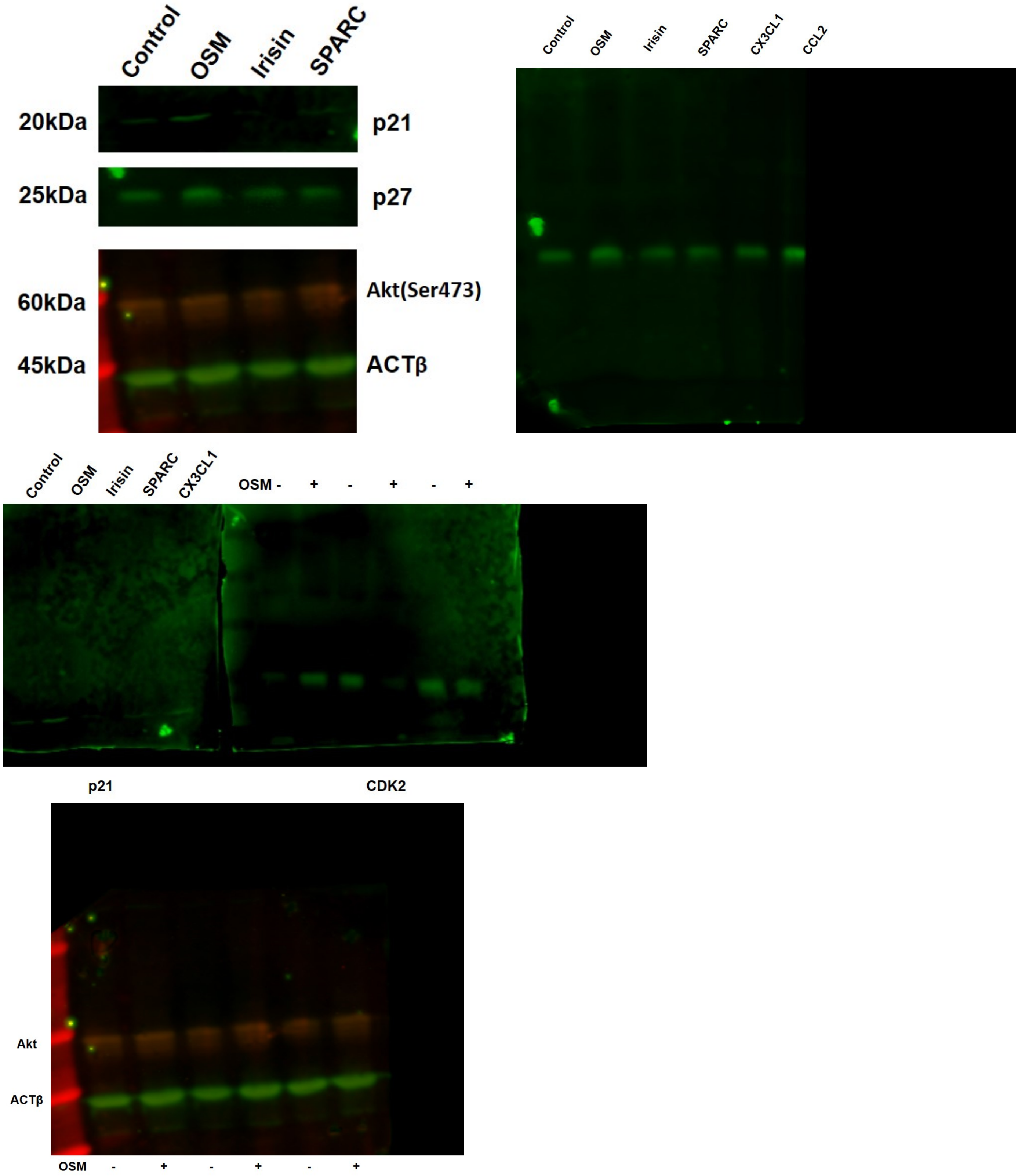

Figure S2. Corresponds to the p21 and p27 bands in Figure 6.

Table S1. Source data for Figure 6.

**Target**	**Integrated Density Control 1**	**Integrated Density OSM 1**	**Integrated Density Control 2**	**Integrated Density OSM 2**	**Integrated Density Control 3**	**Integrated Density OSM 3**
p21	256	387	177	330	245	231
p27	1240	2010	1270	1830	1105	2124
ACTβ (p21)	26000	23700	15950	18800	20500	11280
ACTβ (p27)	5200	4740	3190	3430	3800	3760
Target	Relative Expression Control 1	Relative Expression OSM 1	Relative Expression Control 2	Relative Expression OSM 2	Relative Expression Control 3	Relative Expression OSM 3
p21	0.009846154	0.016329114	0.011097179	0.017553191	0.01195122	0.020478723
p27	0.238461538	0.424050633	0.398119122	0.533527697	0.29078947	0.564893617
